# Response inhibition as a critical executive function in differentiating attention-deficit/hyperactivity disorder from autism spectrum disorder: a comprehensive attention test study

**DOI:** 10.3389/fpsyt.2024.1426376

**Published:** 2024-11-05

**Authors:** Kangto Lee, In Hee Cho, Jeonghoon Park, Hangnyoung Choi, Keun-Ah Cheon

**Affiliations:** ^1^ Department of Child and Adolescent Psychiatry, Severance Hospital, Yonsei University College of Medicine, Seoul, Republic of Korea; ^2^ Institute of Behavioral Science in Medicine, Yonsei University College of Medicine, Yonsei University Health System, Seoul, Republic of Korea

**Keywords:** autism spectrum disorders, ADHD, comorbidity, children, executive function, response inhibition, comprehensive attention test

## Abstract

**Background:**

Autism Spectrum Disorder (ASD) and Attention-Deficit/Hyperactivity Disorder (ADHD) are both associated with impairment in executive function, particularly in complex attention. Although previous studies using clinical assessments have attempted to delineate differences between these disorders, the findings have been inconclusive. Our study aims to elucidate the differences of endophenotype between ASD, ADHD, and their co-occurring condition utilizing a uniform computerized test.

**Methods:**

The study included children diagnosed with ASD, ASD co-occurring with ADHD (ASD+ADHD), or ADHD who completed the comprehensive attention test (CAT) at Severance Hospital between October 2013 to May 2023. We excluded children with intellectual disability and comorbid major psychiatric or neurologic disorders possibly affecting attention measurement. The participants were categorized into three groups for the comparative analysis of CAT measures: (a) ASD (n=112), (b) ASD+ADHD (n=155), and (c) ADHD (n=104). The study also conducted an exploratory analysis utilizing multivariate linear regression analysis to examine the association between the CAT measures and parent-reported scales.

**Results:**

Notably, the ASD+ADHD and ADHD groups exhibited higher frequency of commission errors (CE) and perseveration errors (PE) compared to the ASD group. In the exploratory analysis, a significant negative association was observed between reaction time (RT) and both the social communication questionnaire (SCQ) and the child behavior checklist (CBCL) externalization scores in the ASD+ADHD and ADHD groups. The ASD+ADHD group tended to show higher standard deviation of reaction time (RTSD) compared to the ASD group.

**Conclusions:**

Our findings suggest that impaired response inhibition is more pronounced in ADHD compared to ASD. We propose altered visual attention, reflecting response inhibition, may serve as potential endophenotypic markers differentiating ADHD from ASD in attentional assessment. Elevated RTSD in the ASD+ADHD group demonstrates additive pathology, suggesting that the neurological mechanisms underpinning impaired sustained attention may differ between the two conditions.

## 1 Introduction

Autism Spectrum Disorder (ASD) and Attention-Deficit/Hyperactivity Disorder (ADHD) are distinct neurodevelopmental disorders with overlapping features (1). Both disorders, which are highly heritable, not only share genetic factors (2, 3) but also exhibit overlapping symptoms, often leading to diagnostic confusion. Previous studies showed that 40% to 70% of individuals with ASD not only exhibit symptoms of ADHD but also meet the diagnostic criteria for ADHD (4–6). Similarly, individuals with ADHD commonly present symptoms of ASD. They often display social communication challenges, such as impaired emotion recognition and deficits in pragmatic language skills, compared to typically developing peers (7, 8). This significant overlap in phenomenology complicates diagnosis for clinicians and hinders symptom alleviation and the acquisition of adaptive skills for patients.

Executive function (EF) encompasses a set of cognitive and emotional regulatory functions, including planning, decision-making, and impulse control, facilitating self-regulation and goal achievement (9). EF features in neurodevelopmental conditions like ASD and ADHD have been identified as potential endophenotypes in various studies (10, 11). Both conditions exhibited similar characteristics in overall EF (12–14), prompting research identify EF features that may differentiate ASD and ADHD as potential endophenotypes. For instance, a systematic review of 26 studies identified response inhibition in ADHD and cognitive flexibility and planning in ASD as more impaired in ASD (10). Another review largely agreed, though it associated planning challenges with ADHD rather than ASD (15). However, the most recent meta-analysis argued that no significant differences in EF domain between the two conditions (16), suggesting ongoing controversy. These inconsistency likely stem from small sample sizes in the studies included in the reviews, evolving diagnostic criteria, and heterogeneity of EF measurement tasks between studies (10, 15, 16). In particular, one study utilizing the continuous performance test (CPT) included 19 participants in the ASD group, 29 in the ASD co-occurring with ADHD (ASD+ADHD) group, and 18 in the ADHD group (17), while another study included 9, 11, and 38 participants in these groups, respectively (18). Although, one larger study included 124, 97, and 98 participants in each group, studies of such large sample sizes were uncommon (19). Furthermore, the studies analyzed in meta-analyses used heterogeneous tasks to measure EF domains (10, 15, 16), limiting the generalizability of finding clinical practice.

The comprehensive attention test (CAT) (20, 21) is one of the computerized tests in clinical settings to assess EF. This test evaluates omission errors (OE), commission errors (CE), mean reaction time (RT), and standard deviation of RT (RTSD). In children diagnosed with ADHD, previous study has demonstrated higher OE, CE, and RTSD compared to non-diagnosed children (22). Studies involving children with ASD have reported similar patterns, including higher OE and CE, slower RT, elevated RTSD, and reduced signal detectability (18, 23–25). However, which of these characteristics are specific to each condition remains a matter of debate, likely due to the lack of large-sample clinical studies and limited comparative research among ASD, ADHD, and ASD+ADHD. In this study, we aim to elucidate specific CAT characteristics within clinical groups of ASD, ADHD, and ASD+ADHD to evaluate differences in the EF endophenotypes between these two neurodevelopmental conditions.

## 2 Methods

### 2.1 Participants and psychometric measures

This study utilized retrospective data obtained through a review of electronic medical records of patients treated at the Department of Child and Adolescent Psychiatry at Severance Hospital, Seoul, South Korea. A total of 4,200 patients who underwent the CAT at the clinical psychology department of Severance Hospital from October 2013 to May 2023 were initially screened. A total of 476 patients who had received a diagnosis of ASD, ASD+ADHD, or ADHD based on Diagnostic and statistical manual of mental disorders, 5th edition [DSM-5 (1)] criteria were selected. The final diagnoses of ASD, ASD+ADHD, and ADHD were determined by consensus between the two child and adolescent psychiatrists in the developmental clinic. In the case of ADHD, only patients with the combined presentation were included in the study. Full-scale intelligence quotient (FSIQ) was assessed using the Korean Wechsler intelligence scale for children-IV [K-WISC-IV (26)].

Several parent-reported scales were implemented, including Korean ADHD rating scales [K-ARS (27)] for assessing ADHD symptoms, Korean version of childhood autism rating scale [CARS (28)], and Korean version of social communication questionnaire [SCQ (29)] to evaluate social communication and repetitive, stereotyped patterns of behaviors. Additionally, Korean version of social responsiveness scale [SRS (30)] was used to assess social impairment, and Korean version of child behavior checklist [CBCL (31)] was administrated to evaluate internalizing and externalizing behavioral symptoms.

**Figure 1:**
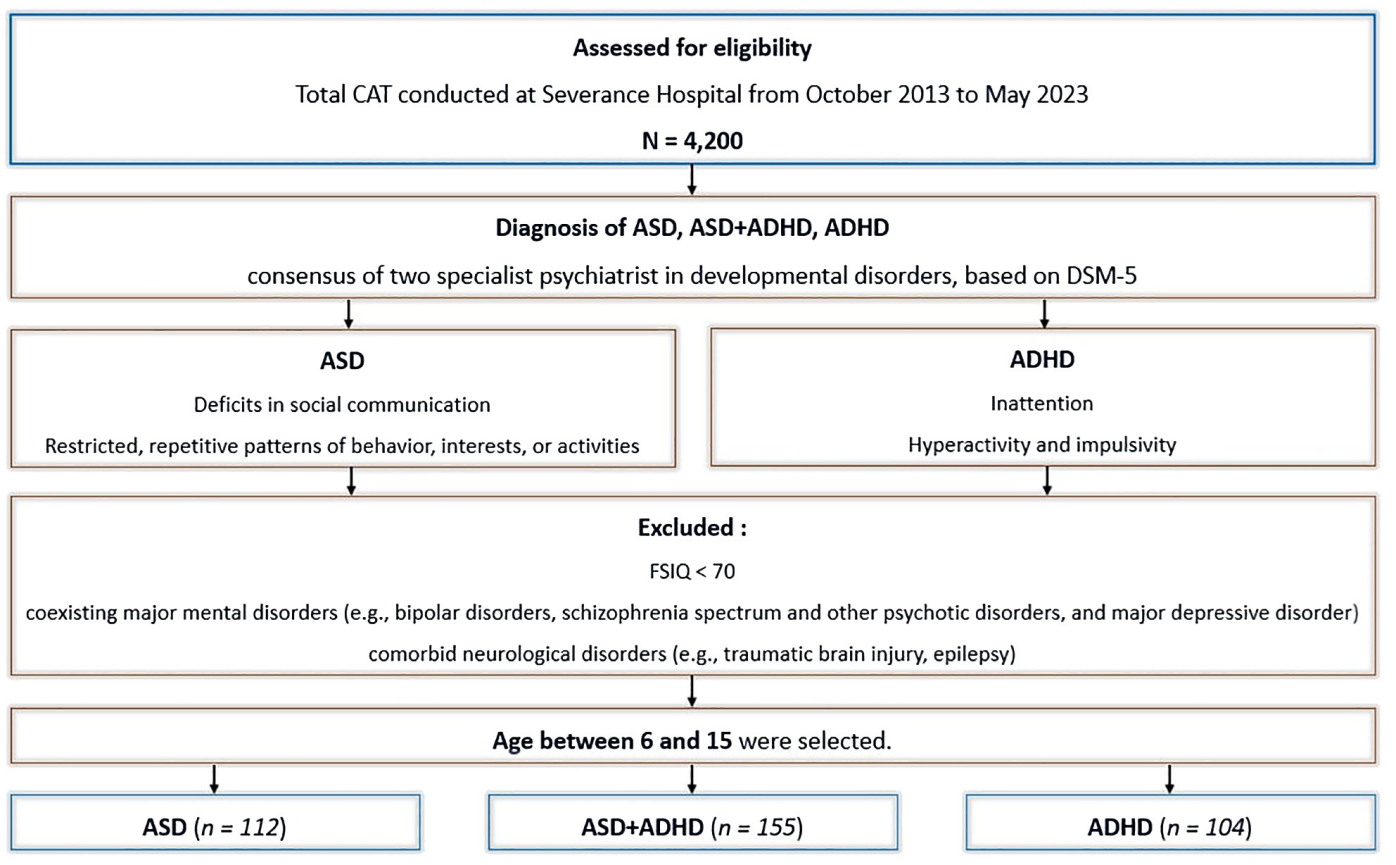
Flowchart of study design and data selection process. ASD, autism spectrum disorder. ADHD, attention-deficit/hyperactivity disorder. ASD+ADHD, ASD and co-occurring ADHD. CAT, comprehensive attention test. DSM-5, Diagnostic and statistical manual of mental disorders, 5th edition. FSIQ, full-scale intelligence quotient.

Patients with coexisting major mental disorders such as bipolar disorders, schizophrenia spectrum and other psychotic disorders, as well as major depressive disorder, intellectual disability, or neurological disorders such as traumatic brain injury and epilepsy, were excluded. Preschool-aged children(under 6 years) were also excluded, as they exhibit considerable behavioral variability, which reduces the test-retest reliability of computerized tests (32). Furthermore, differences in EF tend to become diluted in late adolescence (24). Therefore, only individuals aged 6 to 15 years were included in the analysis, spanning from childhood to middle adolescence. Ultimately, 371 patients were selected for the final analysis. The flowchart depicting data selection process is shown in **Figure 1**.

This study was approved by the Institutional Review Board of the Severance Hospital, Yonsei University, Seoul, Republic of Korea. Informed consent was waived due to the use of retrospective and de-identified patient data (IRB number: 4-2023-1715).

### 2.2 CAT measures

**Figure 2:**
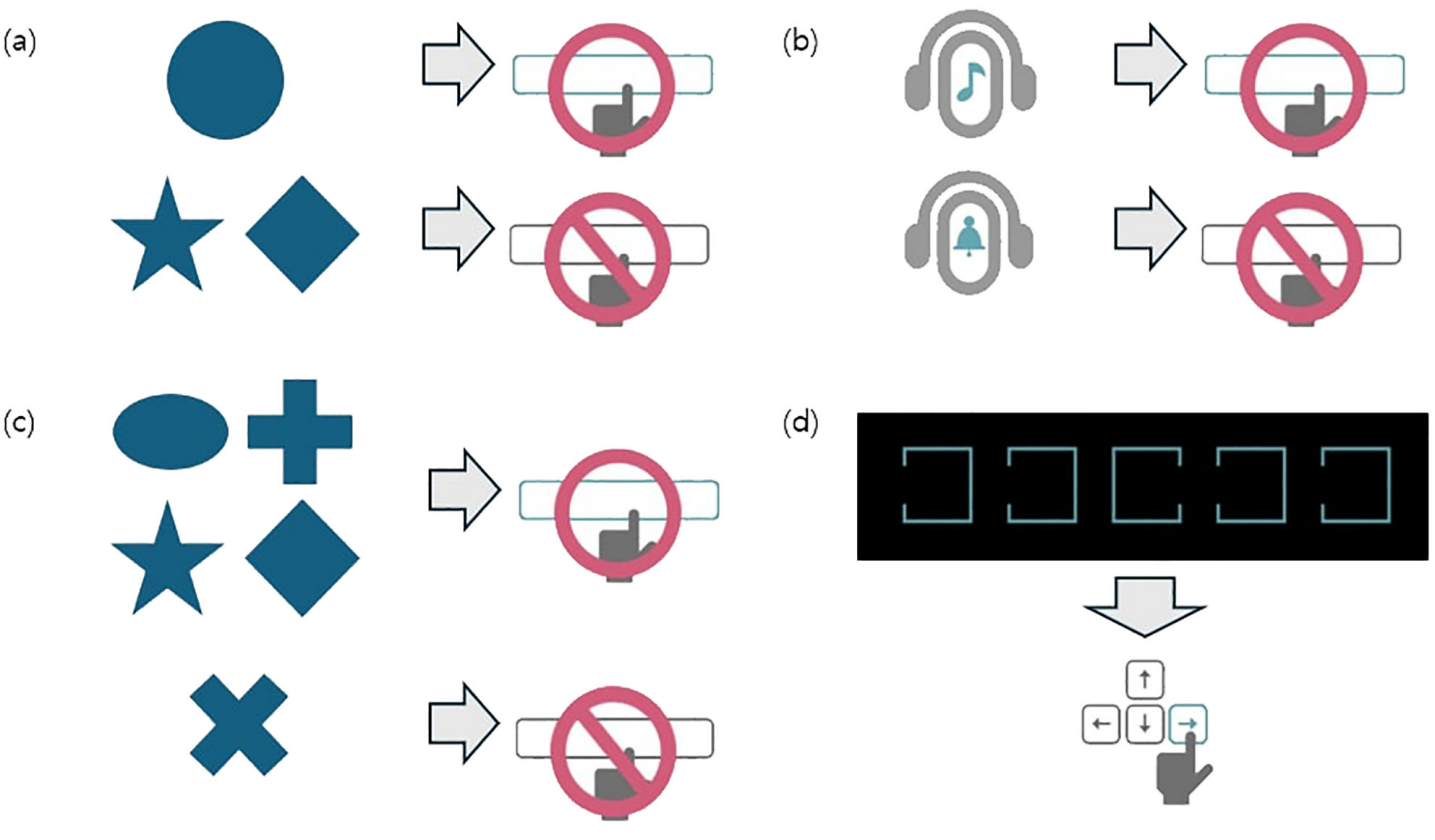
Graphical schema of the comprehensive attention test. Each visual or auditory stimuli was presented on a black screen at 2-second intervals. **(A)** In the visual selective attention test (VA), the patients were instructed to press a button as quickly as possible whenever a circle appeared while ignoring other shapes. A total of 150 stimuli were presented, of which 75 were circles. **(B)** In the auditory selective attention test (AA), the patients were instructed to press a button as quickly as possible upon hearing a specific bell sound, while ignoring other auditory stimuli. A total of 150 stimuli were presented, of which 75 were target sounds. **(C)** In the sustained attention to response task (SAR), the patients were instructed to ignore only the X shapes and press the button as quickly as possible for all other shapes. A total of 300 stimuli were presented, including 75 X shapes. **(D)** In the flanker test (FT), the patients were instructed to press the arrow key (left or right) corresponding to the direction of the open side of the middle box, as quickly as possible, when five boxes with one open side appeared on the screen. A total of 150 stimuli were presented. Adapted with permission from Happymind INC (https://happymindtests.kr/), available at https://quilled-time-26f.notion.site/CAT-Comprehensive-Attention-Test-aa6eb15fbdc04f339b4de937631f0c3a.

CAT data for each participant were collected with four subtests were included in the analysis: Visual selective attention test (VA), Auditory selective attention test (AA), Sustained attention to response task (SAR), and flanker test (FT). In the VA and AA subtests, participants were instructed to press a button as quickly as possible in response to circle-shaped (visual) or bell sound (auditory) stimuli and to withhold responses to other stimuli if other stimuli. Each stimulus was presented at 2-second intervals. In the SAR subtest, participants were instructed to press a button as quickly as possible when non- “X” shapes appeared, and refrain from responding to an “X” shape, similar to the stop signal task, which demands the inhibition of motor responses. In the FT subtest, participants were shown five open boxes and instructed to press the key corresponding to the direction of the open side of the center box as quickly as possible (20). A graphical representation of the CAT is provided in **Figure 2**.

The variables included in the analysis for each subtest were omission error (OE), commission error (CE), reaction time (RT), standard deviation of reaction time (RTSD), and perseveration error (PE). OE and CE refer to the number of omissions and commissions, respectively, with OE primarily reflecting deficits in sustained attention, and CE indicating impulsive responses during rapid information processing, reflecting the degree of response inhibition (33). RT denotes the average time required to respond to the target stimulus, while RTSD serves as an indicator of the consistency of response times to target stimuli. RT is influenced by factors such as inattention and reflects information processing speed (34, 35). RTSD, which captures variability in response speed, reflects impaired attention maintenance across the task (34). Both RT and RTSD are measured in units of milliseconds (ms). PE refers to the number of errors resulting from premature responses within a specific time frame (200ms), indicating impaired response inhibition and compulsive response style.

The CAT has been validated for use in Korean children and adolescents, with multiple studies supporting its efficiency (20, 21). One study assessed test-retest reliability by retesting twenty-one children after a 2-week interval. The results of the paired t-test indicated no significant differences between the initial and retest scores across all subtests, with an average correlation coefficient of.715 (20). Construct validity was verified through principal axis factoring, which revealed that three factors accounted for 51.7% of the total variance of the CAT, demonstrating high validity and reliability (20). Another study examined the diagnostic utility of the CAT compared to the CPT for diagnosing ADHD. The study evaluated the sensitivity and specificity of both tests in 110 children and adolescents. The CPT demonstrated a sensitivity of.419 and a specificity of.806, while the CAT demonstrated a sensitivity of.827 and a specificity of.444. The areas under the receiver operating characteristic curves (AUC) were.664 for the CPT and.692 for the CAT, showing no significant difference between the two. The CAT exhibited moderate specificity and high sensitivity in diagnosing ADHD (21).

### 2.3 Statistical analysis

We used IBM SPSS statistics 26 (SPSS) for the overall statistical analysis. The whole sample was divided into three groups (ASD, ASD+ADHD, and ADHD) as previously described. To assess the normality of the CAT measure for each group, the Kolmogorov-Smirnov test was applied. Given that the variables did not meet the assumption of normality, non-parametric methods were employed. Comparisons of the CAT measures were made using the Kruskal-Wallis test, with the exception of gender distribution, which was assessed using the chi-square test. For variables with statistically significant differences, the Mann-Whitney test was conducted as a *post-hoc* test, with the false discovery rate (FDR) correction applied using the Benjamini-Hochberg method to account for multiple comparisons.

In addition, an exploratory analysis was conducted for each group to examine the association between psychometric variables and CAT measures. Multivariate linear regression analysis was performed, with the independent variables including K-ARS, CARS, SCQ, SRS-total, CBCL-internalization, and CBCL-externalization, while the dependent variables consisted of the CAT measures. Age, sex, and FSIQ were included as control variables. To address multicollinearity, the five subscale scores of the SRS and the CBCL total score were excluded from the independent variables. Since the CAT measure did not meet normality, variables with skewness exceeding 0.5 were square-root transformed for analysis. The FDR correction was again applied using the Benjamini-Hochberg method to account for multiple comparisons.

## 3 Results

### 3.1 Demographic data

A total of 371 participants were eligible for analyses. The participants were divided into three groups: 112 children with ASD (mean age 8.06 (2.13); 93 (83.0%) males), 155 children with ASD+ADHD (mean age 8.12 (2.13); 137 (88.4%) males), 104 children with ADHD (mean age 7.36 (1.66); 82 (78.8%) males). The chi-squared test revealed that no significant difference in gender distribution across the groups. Additionally, no statistically significant difference of FSIQ was observed across groups. However, age distribution was significantly different (p = .043) with the ASD group being significantly older than the ADHD group (p = .012). Detailed demographic data are provided in **Table 1**.

**Table 1:** Demographic and clinical data of each group and pattern of pairwise comparisons.

	ASD, n=112	ASD+ADHD, n=155	ADHD, n=104	p-value of Kruskal-Wallis test *(or Chi-squared test)*	*Post-hoc tests* *(Mann-Whitney)*
Male, *n (%)*	93 (83.0%)	137 (88.4%)	82 (78.8%)	0.112^a^	–
Age, *M (SD)* years	8.06 (2.13)	8.12 (2.13)	7.36 (1.66)	0.043*	ADHD < ASD
FSIQ, *M (SD)*	86.78 (13.62)	87.16 (13.89)	83.33 (14.07)	0.099	–
K-ARS	15.19 (9.01)	19.56 (10.27)	16.32 (9.14)	0.001***	ASD < ASD+ADHD, ADHD
CARS	27.382 (3.43)	25.10 (2.95)	22.26 (2.79)	< 0.001***	ADHD< ASD+ADHD < ASD
SCQ	11.52 (7.85)	10.27 (6.90)	6.63 (5.32)	< 0.001***	ADHD < ASD, ASD+ADHD
SRS-Total score	73.73 (17.65)	71.30 (17.06)	65.42 (17.93)	< 0.001***	ADHD < ASD, ASD+ADHD
SRS-Social awareness	58.15 (12.61)	59.19 (13.50)	54.58 (11.80)	< 0.001**	ADHD < ASD, ASD+ADHD
SRS-Social cognition	65.82 (13.36)	63.36 (13.67)	58.63 (14.32)	< 0.001***	ADHD < ASD, ASD+ADHD
SRS-Social communication	75.67 (17.87)	72.84 (17.29)	68.58 (23.83)	< 0.001***	ADHD < ASD, ASD+ADHD
SRS-Social motivation	66.41 (17.49)	64.16 (16.01)	68.74 (18.93)	0.016*	ASD < ADHD
SRS-Autistic mannerisms	78.83 (20.64)	75.52 (19.70)	64.42 (14.36)	< 0.001***	ADHD < ASD, ASD+ADHD
CBCL-Total score	61.22 (11.69)	63.32 (10.14)	63.32 (13.36)	0.102	–
CBCL-Internalization	59.54 (12.02)	58.83 (10.48)	62.47 (11.15)	0.809	–
CBCL-Externalization	56.40 (10.70)	59.62 (9.91)	57.79 (14.62)	0.005**	ASD< ADHD, ASD+ADHD

a. The p-value of chi-square test was used to find out the difference in gender distribution.

ASD, autism spectrum disorder. ADHD, attention-deficit/hyperactivity disorder. ASD+ADHD, ASD and co-occurring ADHD. M, mean. SD, standard deviation. FSIQ, Full-scale Intelligence Quotient. p-value is calculated by Kruskal-Wallis test. Mann-Whitney test has done as post-hoc test. K-ARS, Korean version of ADHD rating scale. CARS, Childhood Autism Rating Scale. SCQ, Social Communication Questionnaire. SRS, Social Responsiveness scale. CBCL, Child Behavior Checklist.

p-values: *<=.05, **<=.01, ***<=.001.

### 3.2 Parent-report scales

There were significant differences in ASD (CARS, SCQ, SRS) and ADHD symptoms (K-ARS) between the groups. Both the ASD+ADHD and ADHD groups exhibited significantly higher ADHD symptoms compared to the ASD group (p = .001). In contrast, the ASD symptom scales were significantly elevated in the ASD and ASD+ADHD groups compared to the ADHD group, with the exception of the SRS social motivation subcategory. Moreover, CARS, SCQ, and the SRS total score, as well as social awareness, social cognition, and social communication subscales of the SRS, showed significant differences between groups (p <.001 for each of the measure). No statistical differences were found in the CBCL total score or internalizing symptoms, but the ASD+ADHD and ADHD groups reported significantly higher levels of externalizing symptoms compared to the ASD group (p = .005). Detailed information is summarized in **Table 1**.

### 3.3 CAT results

The results of the statistical analysis and mean (standard deviation [SD]) values for the CAT parameters across the three groups are presented in **Table 2**.

**Table 2:** Comparison of CAT parameters between the three groups.

Measures	ASD, n=112	ASD+ADHD, n=155	ADHD, n=104	p-value (Kruskal-Wallis)	*Post-hoc* tests (Mann-Whitney)	p-value (*Post-hoc*)		
						ASD *vs* ASD+ADHD	ASD+ADHD *vs* ADHD	ADHD *vs* ASD
Visual selective attention test (VA)								
OE	5.99 (10.17)	6.92 (9.09)	5.02 (7.84)	0.06	–	–	–	–
CE	8.76 (9.26)	13.83 (12.58)	12.47 (10.05)	0.002**	ASD < ASD+ADHD, ADHD	0.003	0.945	0.002
RT	597.47 (169.07)	582.65 (143.82)	545.93 (132.82)	0.033*	ADHD < ASD+ADHD	0.780	0.024	0.019
RTSD	186.52 (74.18)	217.2 (98.3)	188.45 (93.02)	0.038*	n.s.	0.051	0.023	0.558
PE	1.53 (4.1)	4.02 (7.69)	3.72 (8.97)	0.003**	ASD < ASD+ADHD, ADHD	0.001	0.372	0.024
Auditory selective attention test (AA)								
OE	5.61 (9.16)	7.36 (9.81)	6.28 (8.65)	0.071	-	–	–	–
CE	7.55 (10.21)	12.23 (14.44)	10.31 (9.49)	0.002**	ASD < ASD+ADHD, ADHD	0.007	0.595	0.001
RT	865.85 (245.59)	923.27 (264.17)	877.53 (249.15)	0.265	-	–	–	–
RTSD	260.71 (102.74)	291.45 (123.17)	268.47 (92.32)	0.047*	ASD < ASD+ADHD	0.015	0.234	0.208
PE	3.95 (7.97)	5.52 (9.95)	4.29 (7.39)	0.161	-	–	–	–
Sustained attention to response task (SAR)								
OE	23.95 (38.25)	32.13 (39.86)	24.93 (29.36)	0.011*	ASD < ASD+ADHD	0.003	0.223	0.107
CE	21.8 (15.11)	25.88 (16.43)	27.34 (14.3)	0.009**	ASD < ADHD	0.036	0.286	0.002
RT	660.8 (178.94)	646.31 (156.39)	623.57 (138.65)	0.298	–	–	–	–
RTSD	229.28 (83.84)	260.02 (95.91)	252.72 (97.78)	0.039*	ASD < ASD+ADHD	0.012	0.585	0.082
PE	9.37 (20.18)	14.8 (23.41)	13.26 (20.25)	0.026*	ASD < ASD+ADHD, ADHD	0.015	0.997	0.022
Flanker test (FT)								
OE	19.91 (21.61)	25.89 (28.81)	22.78 (22.98)	0.58	–	–	–	–
CE	31.39 (21.77)	26.07 (19.11)	27.97 (16.21)	0.152	–	–	–	–
RT	813.13 (240.05)	799.78 (221.07)	753.41 (208.06)	0.223	–	–	–	–
RTSD	290.66 (124.63)	298.42 (127.49)	287.05 (125.5)	0.901	–	–	–	–
PE	8.18 (12.5)	7.06 (10.94)	7.99 (12.76)	0.454	–	–	–	–

Significant results in the post-hoc analysis using the Benjamini-Hochberg method were highlighted. ASD, autism spectrum disorder. ADHD, attention-deficit/hyperactivity disorder. ASD+ADHD, ASD and co-occurring ADHD. OE, omission error. CE, commission error. RT, reaction time. RTSD, standard deviation of RT. PE, perseveration error.

n.s., not significant in post-hoc test. p-values: n.s.>.05; *<=.05, **<=.01.

#### 3.3.1 Visual selective attention test

The comparison of OE among the three groups did not reveal significant differences. However, both the ASD+ADHD and ADHD groups exhibited significantly higher CE compared to the ASD group (p = .003,.002, respectively). The ASD+ADHD group showed delayed RT compared to ADHD group (p = 0.024). While the comparison of RTSD showed significant differences in mean comparison (p =.038), the *post-hoc* tests did not yield statistically significant results with marginal p-values. Additionally, both the ASD+ADHD and ADHD groups produced significantly more PE compared to the ASD group (p = .001,.024, respectively).

#### 3.3.2 Auditory selective attention test

No significant differences were observed among the three groups in terms of OE, RT, and PE. However, both the ASD+ADHD and ADHD groups exhibited significantly higher CE than the ASD group (p = .007,.001, respectively). The RTSD for the ASD+ADHD group was significantly higher than that of the ASD group (p = .015), although the ADHD group did not show significant differences in RTSD compared to the other two groups.

#### 3.3.3 Sustained attention to response task

The ASD+ADHD group made significantly more OE than the ASD group (p = .003), while the ADHD group produced more CE compared to the ASD group (p = .002). Additionally, the RTSD for the ASD+ADHD group was significantly higher than that of the ASD group (p = .012). The ASD group produced fewer PE compared to the ASD+ADHD and ADHD groups (p = .015,.022, respectively). There were no significant differences in RT among the three groups.

#### 3.3.4 Flanker test

The comparisons among the three groups did not reveal any significant differences in OE, CE, RT, RTSD, or PE for the flanker test.

#### 3.3.5 Association between psychometric variables and CAT measures

In the exploratory analysis, no significant associations were found between the psychometric variables and the CAT measures in the ASD group. However, in the ASD+ADHD group, the SCQ had a significant negative effect on the RT of the AA (R² = .187, β = -.451, p <.001). Furthermore, the CBCL-externalization score also had a negative effect on the RT of SAR and FT (R² = .383 and.405, respectively; β = -.293 and -.327, respectively; p = .004 and.001, respectively).

In the ADHD group, K-ARS had significant positive association with the RT of VA (R² = .923, β = .680, p = .001), while SCQ and CBCL-externalization scores showed significant negative associations with the same dependent variable (β = -.656, and -0.801, respectively; p = .001, and.003, respectively). Additionally, both SCQ and CBCL-externalization scores had significant negative effects on the RTSD of AA (R² = .922, β = -.641 and -.949, respectively; p = .002 and.001, respectively). The results of the multivariate linear regression analysis are presented in **Supplementary Table S1** through **Supplementary Table S3**.

## 4 Discussion

We analyzed the clinical scales and comprehensive attention test (CAT) measures across different patient groups. The ASD+ADHD group represented greater omission errors (OE) than ASD group in sustained attention to response task (SAR). Both the ASD+ADHD and ADHD groups produced more commission errors (CE) than ASD group in visual selective attention test (VA) and auditory selective attention test (AA). The ADHD group also demonstrated more CE than ASD group in SAR. Additionally, the ASD+ADHD group showed significantly higher reaction time (RT) values than the ADHD group in VA. The ASD+ADHD group also tended to show higher standard deviation of reaction time (RTSD) in AA and SAR. Both ASD+ADHD and ADHD groups produced more perseveration error (PE) in VA and SAR compared to the ASD group. No significant differences were observed in the flanker test (FT) among the groups. In the exploratory analysis, psychometric variables in the ASD group did not have a significant effect on the CAT measures. In the ASD+ADHD group, the SCQ score showed a significant negative association on the RT of AA. In addition, the CBCL-externalization score exhibited significant negative association on the RT of both SAR and FT. In the ADHD group, K-ARS had a positive association on the RT of VA, and SCQ and CBCL-externalization scores had significant negative associations on both the RT of VA and the RTSD of AA.

Our finding showed distinct differences in CE among the groups. These errors, which arise from impulsive information processing, are linked to deficits in sustained attention and response inhibition (33, 36). While some studies suggest that the differences in CE between ASD and ADHD are not significant (17, 18, 37, 38), others align with our findings, indicating that both the ASD+ADHD and ADHD groups exhibit more CE than the ASD group (10, 39). In our study, with the exception of the FT, both the ASD+ADHD and ADHD groups consistently showed a greater tendency to commit CE than the ASD group. No significant differences were noted between the ASD+ADHD and ADHD groups, consistent with previous findings (17, 18, 37, 38). Our findings suggest that elevated CE, often associated with impaired response inhibition, is more pronounced in ADHD compared to ASD, though it may also be present in ASD to a lesser degree (40). One large study on ASD patients found that severe ADHD symptoms predicted increased CE, while ASD symptoms did not correlate with CE in continuous performance test (36). Our study supports such finding, proposing that CE serves as an endophenotypic marker differentiating ADHD from ASD.

PE was another area of interest. PE refers to errors made by responding within 200ms of a stimulus being presented, indicating motor responses initiated before fully discerning the stimulus. Therefore, PE is distinguished from CE in that it represents an error where the patient reflexively responds as soon as the presence of a stimulus is perceived (41, 42). PE, like CE, is associated with impaired response inhibition but is more reflexive in nature. More than half of the participants in our study made no PE during the VA test, but those in the ASD+ADHD and ADHD groups produced more PE in the VA and SAR tests, which assess visual stimuli, compared to the ASD group. This suggests that, like CE, high PE is indicative of impaired response inhibition and serves as a characteristic feature that distinguishes ADHD from ASD. Interestingly, no differences in PE were observed among the groups in the AA, suggesting that children with ADHD may respond more reflexively to visual stimuli than to auditory ones. This finding is supported by existing meta-analyses and studies that demonstrate greater attentional impairment in visual tasks compared to auditory tasks for individuals with ADHD (43–45). From our observations of CE and PE, we propose that both ADHD and ASD+ADHD exhibit significant impairments in response inhibition, potentially reflecting shared neurobiological mechanisms. RTSD, an important measures for evaluating sustained attention and nervous system stability (46), has been debated either as a distinguishing feature of ADHD (18, 19, 46–48) or a commonality between ASD and ADHD (49–51). In our study, we observed a trend of increased RTSD in ASD+ADHD patients compared to ASD-only patients. This suggests that additive pathology may manifest in sustained attention when ASD and ADHD co-occur.

In the exploratory analysis, higher SCQ or CBCL-externalization scores in the ASD+ADHD and ADHD groups were associated with faster RT. A high SCQ indicates social difficulties, and a high CBCL-externalization score indicates externalizing behavior, both of which are associated with impaired response inhibition in ADHD (52, 53). Reduced RT resulting from impulsive response initiation is related to deficits in response inhibition. Therefore, the association of social difficulties and externalizing behaviors with faster RT in ADHD can be attributed to the shared underlying factor of impaired response inhibition. However, this association was not found in the ASD group. This discrepancy can be attributed to the finding that, unlike in ADHD, deficits in cognitive flexibility may be more prominent in the EF domain in ASD, and these deficits are less associated with RT (10). Moreover, social difficulties and externalizing behavior in ASD are known to be associated with impaired cognitive flexibility (54, 55). Therefore, it can be inferred that in ASD, deficits in cognitive flexibility, rather than response inhibition, may be associated with higher SCQ or CBCL-externalization score, which are not significantly related to RT. In summary, this exploratory study highlights impaired response inhibition as a key EF feature in ADHD. While the findings suggest that cognitive flexibility may be a significant EF characteristic in ASD, our study provides only indirect evidence for this conclusion. Interestingly, in the ADHD group, K-ARS scores were associated with increased RT in the VA task. This suggests that higher K-ARS scores may be associated with slower responses due to inattention, with this effect being most pronounced in visual tasks. In the ADHD group, SCQ and CBCL-externalization scores showed a negative association with RTSD of AA. This result contrasts with the typical ADHD pattern of increased reaction time variability in attentional tasks, which may be explained by differences in sensory processing. Some individuals with ADHD, especially those with ASD traits, may be hyper-responsive to auditory stimuli, leading to more consistent attention and reduced RTSD (56). Additionally, this subgroup may regulate arousal more effectively during auditory tasks, resulting in more stable performance (57).

In this study, the proportion of males was 83.0% in the ASD group and 88.4% in the ASD+ADHD group, which is higher than in previous studies reporting a male-to-female ratio of approximately 3:1 for autism (58, 59). This discrepancy likely stems from the study being conducted at a tertiary hospital, where males are more prevalent in cases of autists with more difficulties (59). The ASD+ADHD group did not show significant age differences compared to the other two groups, although the ASD group was significantly older than the ADHD group. This age difference may be attributed to the tendency for children with higher externalizing symptoms to undergo attention tests at an earlier age, as ADHD children in this study had higher CBCL-externalization scores. In terms of parent-report scales, few children in the ADHD group exceeded the cutoff scores on the CARS, SCQ, and SRS, while few children in the ASD group exceeded the K-ARS cutoff score, indicating the clinical overlap between ASD and ADHD. Overall, the ASD+ADHD and ADHD groups showed higher K-ARS scores, while the ASD and ASD+ADHD groups showed higher scores on the CARS, SCQ, and SRS scales.

Several limitations should be considered when interpreting this study. First, the cross-sectional design limits causal inferences. The study relied on a single administration of the CAT, conducted at one point in time. As with other neuropsychological tests, CAT performance can be influenced by external factors such as environmental conditions, stress levels, and time of day, which were not controlled in this study. Second, the data were collected from a single urban tertiary hospital, limiting the generalizability of the results to rural areas or the broader population. The higher proportion of males in the ASD and ASD+ADHD groups compared to the general population also challenges generalization. Third, minor but statistically significant age differences between groups may have influenced the results. Fourth, since this study did not include typically developing children as a control group, there are limitations in generalizing the differences observed between the groups as unique features of the respective conditions. Without a control group, we cannot definitively determine whether the observed impairments in response inhibition are unique to ADHD or if they are also present, albeit to a lesser degree, in ASD. Future studies should include a control group to better contextualize the EF impairments observed in clinical populations. Lastly, the exclusion of individuals with intellectual disabilities (FSIQ < 70) further limits the generalizability of the findings. Nonetheless, the study utilized a large sample size, enhancing its statistical power and mitigating some limitations.

Despite these limitations, this study is significant due to its large sample size and single-test design, which enhances its statistical power. Computerized tests, such as the CAT, are widely used in clinical settings to assess executive function (EF), providing this study with substantial clinical relevance. Evaluating a patient’s EF is crucial not only for differentiating ASD, ADHD, and ASD+ADHD diagnostically but also for guiding intervention strategies and predicting pharmacological responses. Appropriate psychopharmacotherapy not only alleviates symptoms in ADHD patients (60) but also improves various EF domains (61). In contrast, while pharmacotherapy with stimulants or other non-stimulant ADHD medications is often used to manage ADHD symptoms in ASD, it tends to be less effective than in ADHD patients and may even lead to paradoxical or undesirable effects in the ASD population (62, 63).

Given these considerations, the results of this study suggest that clinicians can use the CAT or other computerized tests to gain more comprehensive insights into a patient’s EF profile. For instance, if a child with ASD demonstrates high CE or PE on subtests using visual stimuli, relative to their overall level of impairment, it can be inferred that this child has more pronounced impairments in response inhibition to visual stimuli compared to other EF domains. In such cases, combining these results with a detailed clinical history may aid in diagnosing ADHD or predicting the efficacy of stimulant medications. Conversely, if an autistic child shows only mildly elevated CE but greater OE, it is important to consider the contribution of autism traits rather than co-occurring ADHD in explaining the overall attention difficulties. It is important to note, however, that the CAT is not a diagnostic tool and can be influenced by factors beyond EF, with only moderate specificity. Therefore, its results should be interpreted as part of a comprehensive clinical evaluation.

Our study was conducted using clinical data from computerized tests administered to patients at a tertiary hospital. We analyzed the clinical characteristics and differences in endophenotypes among the three diagnostic groups. These findings, along with evidence from previous functional magnetic resonance imaging (fMRI) studies, allow us to infer differences in the underlying neurobiological mechanisms. For instance, the neural network responsible for response inhibition is predominantly modulated by the right inferior frontal cortex (r-IFC) and associated co-activated brain regions (64, 65). This network has been shown to be impaired in patients with ADHD (66, 67), while no significant differences were observed between children on the autism spectrum and typically developing children (68). Based on this, we hypothesize that impairment in the r-IFC-related network may result in decreased response inhibition in ADHD, but not in ASD, leading to the higher commission errors (CE) observed in the CAT among ADHD patients in our study. However, as our study did not incorporate neuroimaging, future research could focus on validating the hypothesized mechanisms between neurobiological markers and endophenotypes. Additionally, in our study, the CAT was administered to patients who were not receiving medication. Future research could further investigate executive function (EF) through computerized tests, comparing performance based on the presence or absence of medication within each clinical group.

## 5 Conclusions

To our best knowledge, this is the first study to examine endophenotypes of ASD, ADHD and co-occurring condition using parameters derived from an identical psychological task in a large clinical sample. In summary, we propose response inhibition serves as a promising endophenotype that distinguishes ADHD or comorbidity from ASD. This is evidenced by higher CE and PE on the CAT, with PE being more pronounced in visual stimuli tasks compared to auditory tasks. Furthermore, we suggest that the decline in sustained attention observed in ASD+ADHD, reflected by increased RTSD, reveals an additive pathological effect when both conditions co-exist.

The exploratory analysis further suggested that social difficulties and externalizing symptoms are associated with impaired response inhibition in ADHD, whereas in ASD, these difficulties appear to be less related to response inhibition and may instead be more closely linked to cognitive inflexibility or other distinct features. Future studies should aim to integrate these endophenotypic findings with genetic and neurobiological markers in a prospective setting. A deeper understanding of these endophenotypes may enhance our comprehension of underlying pathologies of both conditions, ultimately leading to more accurate diagnoses and treatments.

## Data Availability

The raw data supporting the conclusions of this article will be made available by the authors, without undue reservation.
