# Supplementary material for: Response inhibition as a critical executive function in differentiating attention-deficit/hyperactivity disorder from autism spectrum disorder: a comprehensive attention test study
Source: Front Psychiatry. 2024 Nov 5;15:1426376. doi: 10.3389/fpsyt.2024.1426376 (PMC11574416; doi:10.3389/fpsyt.2024.1426376)
Supplement: Supplementary file 1 [file Table1.docx]

Supplementary Material

**Supplementary Table 1.**  **Multivariate Linear Regression Analysis of the ASD Group.**

|  | **R²** | **p-value** | **K-ARS**  *β (p-value)* | **CARS**  *β (p-value)* | **SCQ**  *β (p-value)* | **SRS-Total score**  *β (p-value)* | **CBCL-Internalization**  *β (p-value)* | **CBCL-Externalization**  *β (p-value)* |
| --- | --- | --- | --- | --- | --- | --- | --- | --- |
| **Visual selective attention test (VA)** | | | | | | | | |
| OE | 0.700 | 0.112 | -0.077 (0.802) | -0.109 (0.705) | 0.464 (0.140) | 0.399 (0.329) | -0.235 (0.579) | -0.709 (0.107) |
| CE | 0.526 | 0.440 | -0.339 (0.387) | -0.460 (0.220) | 0.219 (0.558) | -0.396 (0.437) | 0.327 (0.540) | 0.628 (0.239) |
| RT | 0.923 | < 0.001 | 0.680 (0.001) | 0.190 (0.211) | -0.656 (0.001) | 0.488 (0.035) | -0.220 (0.315) | -0.801 (0.003) |
| RTSD | 0.743 | 0.065 | 0.000 (1.000) | -0.114 (0.669) | 0.054 (0.844) | 0.373 (0.324) | -0.468 (0.246) | -0.316 (0.411) |
| PE | 0.538 | 0.413 | -0.391 (0.317) | -0.025 (0.943) | -0.035 (0.923) | -0.306 (0.54) | 0.076 (0.884) | 0.376 (0.464) |
| **Auditory selective attention test (AA)** | | | | | | | | |
| OE | 0.777 | 0.038 | -0.012 (0.964) | -0.133 (0.592) | 0.169 (0.512) | 0.478 (0.186) | -0.166 (0.648) | -0.890 (0.028) |
| CE | 0.612 | 0.253 | 0.069 (0.843) | -0.690 (0.057) | 0.160 (0.636) | 0.316 (0.490) | 0.232 (0.628) | -0.637 (0.191) |
| RT | 0.612 | 0.254 | 0.829 (0.036) | 0.055 (0.865) | -0.778 (0.041) | 0.294 (0.521) | 0.291 (0.547) | -0.764 (0.124) |
| RTSD | 0.922 | < 0.001 | 0.502 (0.009) | -0.281 (0.079) | -0.641 (0.002) | 0.552 (0.021) | 0.326 (0.152) | -0.949 (0.001) |
| PE | 0.588 | 0.303 | 0.033 (0.926) | -0.409 (0.241) | -0.396 (0.269) | 0.683 (0.166) | -0.230 (0.642) | -0.498 (0.311) |
| **Sustained attention to response task (SAR)** | | | | | | | | |
| OE | 0.500 | 0.500 | -0.012 (0.977) | 0.116 (0.754) | -0.317 (0.413) | -0.066 (0.898) | 0.108 (0.842) | 0.050 (0.924) |
| CE | 0.582 | 0.315 | 0.371 (0.317) | -0.684 (0.067) | 0.095 (0.785) | -0.210 (0.657) | 0.146 (0.768) | 0.090 (0.852) |
| RT | 0.765 | 0.047 | 0.160 (0.558) | 0.226 (0.382) | -0.172 (0.515) | 0.475 (0.199) | -0.018 (0.962) | -0.491 (0.194) |
| RTSD | 0.700 | 0.111 | 0.247 (0.427) | -0.367 (0.219) | -0.183 (0.538) | 0.034 (0.932) | 0.221 (0.600) | -0.334 (0.420) |
| PE | 0.475 | 0.557 | 0.170 (0.675) | -0.342 (0.377) | -0.229 (0.560) | -0.182 (0.730) | 0.078 (0.888) | -0.055 (0.919) |
| **Flanker test (FT)** | | | | | | | | |
| OE | 0.675 | 0.201 | -0.271 (0.409) | -0.144 (0.667) | 0.076 (0.816) | 0.103 (0.805) | 0.235 (0.600) | -0.185 (0.664) |
| CE | 0.489 | 0.596 | -0.128 (0.751) | -0.050 (0.904) | 0.250 (0.544) | -0.231 (0.658) | 0.599 (0.299) | 0.357 (0.507) |
| RT | 0.796 | 0.047 | 0.187 (0.471) | -0.293 (0.286) | 0.017 (0.947) | 0.344 (0.312) | 0.255 (0.476) | -0.531 (0.141) |
| RTSD | 0.782 | 0.059 | -0.065 (0.805) | 0.000 (1.000) | -0.019 (0.942) | -0.137 (0.689) | 0.201 (0.584) | 0.278 (0.432) |
| PE | 0.562 | 0.431 | -0.193 (0.608) | 0.527 (0.198) | 0.145 (0.702) | -0.571 (0.256) | -0.506 (0.341) | 0.849 (0.112) |

Note: Multivariate linear regression analysis was conducted. False discovery rate (FDR) correction was applied for multiple comparisons. Sex, age and full-scale intelligence quotient were included as control variables. The dependent variables, excluding RT of AA, RT of SAR, and CE, RT, and RTSD of FT, exhibited skewness above 0.5 and were analyzed after applying a square-root transformation. ASD = autism spectrum disorder. K-ARS = Korean version of ADHD rating scale. CARS = Childhood Autism Rating Scale. SCQ = Social Communication Questionnaire. SRS = Social Responsiveness scale. CBCL = Child Behavior Checklist. OE = omission error. CE = commission error. RT = reaction time. RTSD = standard deviation of RT. PE = perseveration error. β = standardized coefficient of the variable in multivariate linear regression analysis.

**Supplementary Table 2.**  **Multivariate Linear Regression Analysis of the ASD+ADHD Group.**

|  | **R²** | **p-value** | **K-ARS**  *β (p-value)* | **CARS**  *β (p-value)* | **SCQ**  *β (p-value)* | **SRS-Total score**  *β (p-value)* | **CBCL-Internalization**  *β (p-value)* | **CBCL-Externalization**  *β (p-value)* |
| --- | --- | --- | --- | --- | --- | --- | --- | --- |
| **Visual selective attention test (VA)** | | | | | | | | |
| OE | 0.392 | < 0.001 | 0.234 (0.021) | -0.011 (0.898) | 0.256 (0.021) | -0.212 (0.105) | -0.223 (0.040) | 0.098 (0.318) |
| CE | 0.134 | 0.031 | -0.049 (0.686) | -0.094 (0.356) | 0.069 (0.598) | 0.110 (0.479) | -0.172 (0.184) | 0.178 (0.130) |
| RT | 0.408 | < 0.001 | 0.243 (0.017) | -0.053 (0.527) | -0.008 (0.941) | 0.103 (0.429) | -0.150 (0.162) | -0.152 (0.117) |
| RTSD | 0.476 | < 0.001 | 0.157 (0.100) | -0.01 (0.900) | -0.021 (0.836) | 0.115 (0.349) | -0.256 (0.012) | -0.001 (0.987) |
| PE | 0.240 | < 0.001 | 0.146 (0.196) | -0.067 (0.481) | 0.111 (0.368) | -0.034 (0.816) | -0.289 (0.018) | 0.136 (0.215) |
| **Auditory selective attention test (AA)** | | | | | | | | |
| OE | 0.339 | < 0.001 | 0.096 (0.359) | -0.054 (0.545) | -0.039 (0.731) | -0.056 (0.678) | -0.029 (0.794) | -0.143 (0.163) |
| CE | 0.198 | < 0.001 | -0.035 (0.763) | -0.093 (0.345) | 0.029 (0.817) | 0.061 (0.684) | -0.053 (0.671) | -0.052 (0.646) |
| RT | 0.187 | 0.002 | 0.092 (0.435) | -0.058 (0.557) | -0.451 (0.001) | 0.399 (0.010) | -0.089 (0.475) | -0.036 (0.747) |
| RTSD | 0.415 | < 0.001 | 0.060 (0.547) | -0.090 (0.285) | -0.170 (0.120) | 0.225 (0.084) | -0.210 (0.049) | -0.110 (0.252) |
| PE | 0.309 | < 0.001 | 0.020 (0.851) | -0.109 (0.230) | 0.033 (0.781) | 0.119 (0.39) | -0.116 (0.314) | -0.156 (0.137) |
| **Sustained attention to response task (SAR)** | | | | | | | | |
| OE | 0.295 | < 0.001 | 0.228 (0.038) | -0.144 (0.124) | 0.112 (0.349) | -0.139 (0.331) | -0.075 (0.528) | -0.148 (0.165) |
| CE | 0.102 | 0.151 | -0.103 (0.405) | -0.207 (0.051) | -0.005 (0.969) | 0.233 (0.150) | -0.024 (0.855) | 0.113 (0.349) |
| RT | 0.383 | < 0.001 | 0.265 (0.012) | -0.079 (0.371) | -0.031 (0.783) | 0.091 (0.500) | -0.016 (0.886) | -0.293 (0.004) |
| RTSD | 0.382 | < 0.001 | 0.069 (0.505) | -0.154 (0.081) | 0.193 (0.090) | 0.064 (0.637) | -0.184 (0.099) | -0.029 (0.768) |
| PE | 0.154 | 0.015 | -0.032 (0.787) | -0.150 (0.144) | 0.137 (0.296) | 0.147 (0.348) | -0.295 (0.024) | 0.161 (0.169) |
| **Flanker test (FT)** | | | | | | | | |
| OE | 0.386 | < 0.001 | 0.121 (0.241) | -0.146 (0.096) | 0.192 (0.088) | -0.174 (0.201) | -0.233 (0.044) | -0.055 (0.579) |
| CE | 0.217 | < 0.001 | 0.088 (0.449) | -0.074 (0.455) | -0.011 (0.930) | 0.104 (0.496) | 0.013 (0.923) | 0.061 (0.590) |
| RT | 0.405 | < 0.001 | 0.211 (0.043) | -0.057 (0.509) | -0.046 (0.677) | 0.150 (0.270) | -0.055 (0.627) | -0.327 (0.001) |
| RTSD | 0.419 | < 0.001 | 0.035 (0.735) | -0.114 (0.183) | 0.064 (0.560) | 0.135 (0.315) | -0.205 (0.069) | -0.097 (0.322) |
| PE | 0.273 | < 0.001 | 0.035 (0.758) | -0.225 (0.019) | 0.309 (0.013) | 0.053 (0.717) | -0.171 (0.172) | -0.033 (0.759) |

Note: Multivariate linear regression analysis was conducted. False discovery rate (FDR) correction was applied for multiple comparisons. Sex, age and full-scale intelligence quotient were included as control variables. The dependent variables, excluding RT of AA, CE and RTSD of SAR, and RT and RTSD of FT, exhibited skewness above 0.5 and were analyzed after applying a square-root transformation. ASD = autism spectrum disorder. ADHD = attention-deficit/hyperactivity disorder. ASD+ADHD = ASD and co-occurring ADHD. K-ARS = Korean version of ADHD rating scale. CARS = Childhood Autism Rating Scale. SCQ = Social Communication Questionnaire. SRS = Social Responsiveness scale. CBCL = Child Behavior Checklist. OE = omission error. CE = commission error. RT = reaction time. RTSD = standard deviation of RT. PE = perseveration error.

**Supplementary Table 3.**  **Multivariate Linear Regression Analysis of the ADHD Group.**

|  | **R²** | **p-value** | **K-ARS**  *β (p-value)* | **CARS**  *β (p-value)* | **SCQ**  *β (p-value)* | **SRS-Total score**  *β (p-value)* | **CBCL-Internalization**  *β (p-value)* | **CBCL-Externalization**  *β (p-value)* |
| --- | --- | --- | --- | --- | --- | --- | --- | --- |
| **Visual selective attention test (VA)** | | | | | | | | |
| OE | 0.700 | 0.112 | -0.077 (0.802) | -0.109 (0.705) | 0.464 (0.140) | 0.399 (0.329) | -0.235 (0.579) | -0.709 (0.107) |
| CE | 0.526 | 0.440 | -0.339 (0.387) | -0.46 (0.220) | 0.219 (0.558) | -0.396 (0.437) | 0.327 (0.540) | 0.628 (0.239) |
| RT | 0.923 | < 0.001 | 0.680 (0.001) | 0.190 (0.211) | -0.656 (0.001) | 0.488 (0.035) | -0.220 (0.315) | -0.801 (0.003) |
| RTSD | 0.743 | 0.065 | 0.000 (1.000) | -0.114 (0.669) | 0.054 (0.844) | 0.373 (0.324) | -0.468 (0.246) | -0.316 (0.411) |
| PE | 0.538 | 0.413 | -0.391 (0.317) | -0.025 (0.943) | -0.035 (0.923) | -0.306 (0.54) | 0.076 (0.884) | 0.376 (0.464) |
| **Auditory selective attention test (AA)** | | | | | | | | |
| OE | 0.777 | 0.038 | -0.012 (0.964) | -0.133 (0.592) | 0.169 (0.512) | 0.478 (0.186) | -0.166 (0.648) | -0.890 (0.028) |
| CE | 0.612 | 0.253 | 0.069 (0.843) | -0.690 (0.057) | 0.160 (0.636) | 0.316 (0.49) | 0.232 (0.628) | -0.637 (0.191) |
| RT | 0.612 | 0.254 | 0.829 (0.036) | 0.055 (0.865) | -0.778 (0.041) | 0.294 (0.521) | 0.291 (0.547) | -0.764 (0.124) |
| RTSD | 0.922 | < 0.001 | 0.502 (0.009) | -0.281 (0.079) | -0.641 (0.002) | 0.552 (0.021) | 0.326 (0.152) | -0.949 (0.001) |
| PE | 0.588 | 0.303 | 0.033 (0.926) | -0.409 (0.241) | -0.396 (0.269) | 0.683 (0.166) | -0.230 (0.642) | -0.498 (0.311) |
| **Sustained attention to response task (SAR)** | | | | | | | | |
| OE | 0.500 | 0.500 | -0.012 (0.977) | 0.116 (0.754) | -0.317 (0.413) | -0.066 (0.898) | 0.108 (0.842) | 0.05 (0.924) |
| CE | 0.582 | 0.315 | 0.371 (0.317) | -0.684 (0.067) | 0.095 (0.785) | -0.210 (0.657) | 0.146 (0.768) | 0.09 (0.852) |
| RT | 0.765 | 0.047 | 0.160 (0.558) | 0.226 (0.382) | -0.172 (0.515) | 0.475 (0.199) | -0.018 (0.962) | -0.491 (0.194) |
| RTSD | 0.700 | 0.111 | 0.247 (0.427) | -0.367 (0.219) | -0.183 (0.538) | 0.034 (0.932) | 0.221 (0.600) | -0.334 (0.420) |
| PE | 0.475 | 0.557 | 0.170 (0.675) | -0.342 (0.377) | -0.229 (0.560) | -0.182 (0.730) | 0.078 (0.888) | -0.055 (0.919) |
| **Flanker test (FT)** | | | | | | | | |
| OE | 0.675 | 0.201 | -0.271 (0.409) | -0.144 (0.667) | 0.076 (0.816) | 0.103 (0.805) | 0.235 (0.600) | -0.185 (0.664) |
| CE | 0.489 | 0.596 | -0.128 (0.751) | -0.050 (0.904) | 0.250 (0.544) | -0.231 (0.658) | 0.599 (0.299) | 0.357 (0.507) |
| RT | 0.796 | 0.047 | 0.187 (0.471) | -0.293 (0.286) | 0.017 (0.947) | 0.344 (0.312) | 0.255 (0.476) | -0.531 (0.141) |
| RTSD | 0.782 | 0.059 | -0.065 (0.805) | 0.000 (1.000) | -0.019 (0.942) | -0.137 (0.689) | 0.201 (0.584) | 0.278 (0.432) |
| PE | 0.562 | 0.431 | -0.193 (0.608) | 0.527 (0.198) | 0.145 (0.702) | -0.571 (0.256) | -0.506 (0.341) | 0.849 (0.112) |

Note: Multivariate linear regression analysis was conducted. False discovery rate (FDR) correction was applied for multiple comparisons. Sex, age and full-scale intelligence quotient were included as control variables. The dependent variables, excluding RT and RTSD of AA, CE and RTSD of SAR, and CE, RT, and RTSD of FT, exhibited skewness above 0.5 and were analyzed after applying a square-root transformation. ADHD = attention-deficit/hyperactivity disorder. K-ARS = Korean version of ADHD rating scale. CARS = Childhood Autism Rating Scale. SCQ = Social Communication Questionnaire. SRS = Social Responsiveness scale. CBCL = Child Behavior Checklist. OE = omission error. CE = commission error. RT = reaction time. RTSD = standard deviation of RT. PE = perseveration error. β = standardized coefficient of the variable in multivariate linear regression analysis.

**Supplementary Table 4.**  **Range of scores for parent-report scales in each group.**

|  | ASD, n=112 | | ASD+ADHD, n=155 | | ADHD, n=104 | |
| --- | --- | --- | --- | --- | --- | --- |
|  | Minimum | Maximum | Minimum | Maximum | Minimum | Maximum |
| K-ARS | 2 | 48 | 1 | 47 | 1 | 46 |
| CARS | 18.5 | 42 | 17.5 | 32 | 18 | 27.5 |
| SCQ | 0 | 38 | 0 | 32 | 1 | 17 |
| SRS-Total score | 32 | 116 | 38 | 124 | 34 | 102 |
| SRS-Social awareness | 33 | 96 | 33 | 100 | 29 | 89 |
| SRS-Social cognition | 43 | 100 | 36 | 102 | 39 | 107 |
| SRS-Social communication | 41 | 124 | 36 | 124 | 33 | 137 |
| SRS-Social motivation | 38 | 111 | 32 | 111 | 35 | 108 |
| SRS-Autistic mannerisms | 41 | 135 | 41 | 126 | 41 | 106 |
| CBCL-Total score | 30 | 90 | 34 | 90 | 40 | 91 |
| CBCL-Internalization | 37 | 93 | 36 | 90 | 38 | 89 |
| CBCL-Externalization | 37 | 90 | 37 | 90 | 31 | 90 |

Note: The minimum and maximum values of the parent-report scale for each group are presented in the table. ASD = autism spectrum disorder. K-ARS = Korean version of ADHD rating scale. CARS = Childhood Autism Rating Scale. SCQ = Social Communication Questionnaire. SRS = Social Responsiveness scale. CBCL = Child Behavior Checklist.
